# Boosting eWOM through Social Media Brand Page Engagement: The Mediating Role of Self-Brand Connection

**DOI:** 10.3390/bs12110411

**Published:** 2022-10-26

**Authors:** Ovidiu-Ioan Moisescu, Oana-Adriana Gică, Flavia-Andreea Herle

**Affiliations:** 1 Department of Marketing, Faculty of Economics and Business Administration, Babeș-Bolyai University, 400591 Cluj-Napoca, Romania; 2 Department of Hospitality Services, Faculty of Business, Babeș-Bolyai University, 400038 Cluj-Napoca, Romania

**Keywords:** electronic word-of-mouth, social media brand page engagement, passive and active engagement, self-brand connection, PLS-SEM, PLSPredict

## Abstract

The present study’s objective is to investigate the influence of active and passive social media brand page engagement on eWOM, via self-brand connection. To accomplish this objective, an online survey was conducted among a sample of Facebook users from Romania, Facebook being the most popular social network worldwide, and Romania being an adequate representation of a European developing country. To assess the proposed research model, we used partial least squares structural equation modeling (PLS-SEM). Our results show that social media brand page engagement, either passive or active, has a positive impact eWOM, both directly and indirectly, via self-brand connection. Additionally, our research reveals that the two types of social media brand page engagement generate eWOM distinctly: although passive engagement has a considerably stronger direct influence on self-brand connection, active engagement is equally influential for both self-brand connection and eWOM. However, due to the mediating role of self-brand connection, the total effect on eWOM is relatively equal for both passive and active engagement. The research provides practical implications for social media marketers, emphasizing the importance of increasing consumers’ engagement with social media brand pages for generating eWOM and, consequently, to attract new customers and to reinforce brand loyalty for existing ones.

## 1. Introduction

Since the early 2000s, the popularity of social media has grown tremendously, the number of social media users worldwide reaching 4.65 billion in 2022, each user spending an average of 147 min on social network platforms daily [1]. Social media platforms enable organizations to engage directly with consumers, and to influence their attitudes and behavior with regard to products and brands. Inherently, marketing spending in social media has grown more than four times since 2017, peaking at 229.5 billion USD in 2022, and being expected to continue to grow to a projected spending of 358 billion USD by 2026 [2].

Social media brand pages are of utmost importance for social media marketers, representing a tool that brands and consumers can use to interact with each other. On such pages, brands communicate information about their products/services, disseminate special offers, provide entertainment, etc., while consumers can virtually dialogue with brands, or contribute with new content [3]. Building strong brands and tight relationships with consumers requires that online marketers stimulate consumers’ engagement with social media brand pages [4,5]. Companies strive for generating, via social media marketing efforts, important volumes of positive electronic word of mouth (eWOM). Researchers and practitioners alike recognize that due to its perceived trustworthiness, credibility, and reliability [6,7], word of mouth, either offline or online, is one of the most important drivers of consumer attitudes and behaviors [8].

Researchers have argued that social media brand page engagement can positively impact eWOM [9,10,11,12,13,14]. However, to establish effective marketing strategies, companies need to gain appropriate knowledge on how social media related interactions could help them acquire and retain customers, and particularly how consumers’ engagement with social media brand pages impacts eWOM.

Previous research on this topic, although extremely valuable, has several limits. Firstly, the conceptual frameworks used by researchers for measuring social media brand page engagement did not enable them to appropriately depict the particular impact of passive engagement (content consumption) and active engagement (content contribution) on eWOM. Secondly, the mediating role of self-brand connection (i.e., the extent to which individuals incorporate brands into their self-concept) has not been investigated in this context. Nevertheless, such a mediating role should be investigated, as it has been empirically proven that consumers’ engagement with brands [15], and particularly their engagement with social media brand pages [16,17,18,19], influences self-brand connection. Additionally, previous research has demonstrated that self-brand connection increases the emotional attachment of consumers to brands, consequently generating word of mouth [20,21,22].

Given such limitations of previous studies, the present paper contributes to extant knowledge by investigating the influence of social media brand page engagement, as consumption (passive engagement) and contribution (active engagement), on eWOM, via self-brand connection, as a crucial mediator of the relationship.

This research is grounded in two theories. Primarily, it applies the social identity theory [23] in a social media context. According to this theory, it should be expected that consumers derive and/or alter their self-concept as a result of their psychological membership in brand-related social media communities. Secondly, based on the cultivation theory [24] in a social media context, it should be assumed that, in time, the contact with brand-related content in social media will modify consumers’ brand perceptions, attitudes, and behaviors.

As Facebook has been the worldwide leader of social media for more than a decade, with approximately 2.9 billion monthly active users in 2022 [25], the current study focuses on this social network platform. Consequently, the present paper aims at investigating the impact of Facebook brand page engagement on eWOM, and the mediating effect of self-brand connection on this relationship. For that, an online survey was conducted among a sample of Facebook users from Romania, Facebook being the most popular social network in the region, and Romania an adequate representation of a European developing country.

## 2. Theoretical Background and Research Hypotheses

### 2.1. Electronic Word of Mouth

According to Hennig-Thurau et al. [26], eWOM consists of statements, either positive or negative, originating from potential, actual or former customers, regarding a brand or a company, when these statements are disseminated to a various group of other individuals or organizations via the Internet. Summarizing, eWOM refers to the online exchange of marketing information between consumers [27]. The types of platforms that allow users to post user-generated content [28] has developed constantly during the last decades, enabling consumers to spread eWOM via blogs, online forums, chat rooms, online shopping sites, but most often via social networking sites.

As researchers and practitioners argue, word of mouth, in both its forms (i.e., offline and online), is perceived as a source of reliable, credible and trustworthy information [6,7], and, as a consequence, has a strong influence on consumer behaviors and attitudes [8].

As previous research underlines, word of mouth can influence consumers’ purchase behavior, their intentions, as well as their attitudes toward brands, enabling them to strengthen their brand choices, and to reduce dissonance. Hence, word of mouth can be considered a powerful tool for attracting new customers and retaining existing ones [29,30].

Compared to offline word of mouth, eWOM, especially if generated through social media, has a faster and larger penetration because individuals tend to have many more friends and acquaintances virtually than they do offline [31].

### 2.2. Self-Brand Connection

Self-brand connection is a reflection of the extent to which individuals include the brand into their self-concept [15,18,32]. For a consumer, a brand is relevant if it shares similar values with that consumer. In other words, self-brand connection represents the connection that consumers make between a brand and their own identity.

Extant literature states that self-brand connection can generate word of mouth by increasing the emotional attachment of customers to brands [20,21,22]. In line with the social identity theory [23], one of the main reasons consumers share brand-related content online is because this represents a form of expressing themselves [33]. Hence, the self-expressive nature of a brand should lead to eWOM diffusion about that particular brand, as this action represents a reflection of consumer identity [20]. Based on such theory, we issued the following research hypothesis:

**H1.**  *Self-brand connection positively influences eWOM*.

### 2.3. Social Media Brand Page Engagement

As stated by Hollebeek et al. [34], engagement represents a psychological disposition of a person to interact with certain objects. Translating this definition in the social media context, engagement with social media brand pages illustrates the willingness of a person to develop a relationship with that particular brand’s hosted social media [3].

According to the consumer’s online brand-related activities (COBRA) framework [4,5], there are two components of engagement with social media brand pages: consumption or passive engagement (consisting in social media page visits, posts reading, pictures or videos viewing, etc.), and contribution or active engagement (referring to commenting on posts, photos or videos, asking questions, engaging in conversations with other members of the social media brand page community, etc.).

In line with the social identity theory [23], it can be argued that, in the context of social media, the psychological membership in social media communities, built around brands, can determine consumers to derive and/or alter their self-concept. Additionally, based on the cultivation theory [24], it can be assumed that, in time, the contact with brand-related content in social media will modify consumers’ brand perceptions, attitudes, and behaviors.

Moreover, it has been empirically proven that consumers’ engagement with brands [15], and particularly with social media brand pages [16,17,18,19], influences self-brand connection. The extent to which customers perceive themselves as being similar to the brand, in terms of attitudes, behaviors and expectations, is driven by the interactions that take place on the brand’s social networks [35]. This similarity enhances consumers’ sense of belonging to the brand’s community, which reflects the consumer’s identification with the brand and its affiliated members [36].

On these grounds, we proposed the following research hypotheses:

**H2a.**  *Passive social media brand page engagement positively influences self-brand connection*.

**H2b.**  *Active social media brand page engagement positively influences self-brand connection*.

Previous studies have suggested that social media brand page engagement can directly and positively impact eWOM [9,14]. Customers’ online activities such as reviewing or giving feedback, as well as their brand-related thoughts posted in social media brand communities, have the ability to generate eWOM via other consumers who engage with that online community [37].

Consumers engaged with a brand community give feedback and share their experiences with other members of the same social circle. Further on, positive experiences with the brand’s virtual communities are likely to determine customers to spread positive word of mouth and recommend the brand to others [38]. Thus, consumers engaged in online communities are likely to advocate brands [39] and show a higher tendency to spread positive word of mouth [40]. Hence, customer engagement is crucial for driving word-of-mouth activities in online brand communities [38].

Considering these arguments, we issued the following research hypotheses:

**H3a.**  *Passive social media brand page engagement positively influences eWOM*.

**H3b.**  *Active social media brand page engagement positively influences eWOM*.

**Figure 1:**
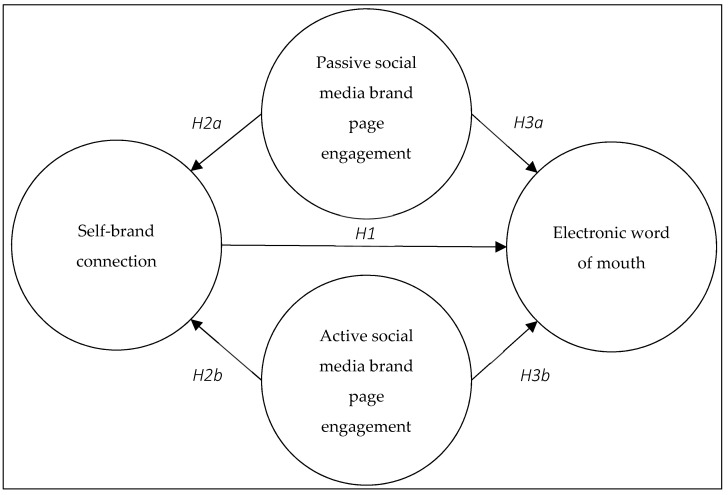
Proposed structural model.

Aggregating the posited research hypotheses, we proposed the structural research model depicted in Figure 1.

## 3. Methodology

### 3.1. Research Objective, Data Collection and Sampling

The objective of the current research was to investigate the influence of active and passive social media brand page engagement on eWOM, via self-brand connection, as a mediator of the relationship. To accomplish this research objective, we conducted an online survey among Facebook users from Romania.

Facebook has been the worldwide leader of social media for more than a decade, being today the most popular social network worldwide with approximately 2.9 billion monthly active users in 2022 [25]. Romania is a developing country, one of the largest in the EU, in terms of population (the 6th out of 27 member countries). With a population of 19 million, Romania had 15.5 million internet users in 2021, and more than 11.6 million social network users, Facebook being the most used social media platform, with 11.4 million active users [41]. 

Our survey targeted active Facebook users who had stated their affinity for a brand by following its Facebook page. Data were collected during February–May 2020. We employed a convenience sampling approach and disseminated the online questionnaire on various Facebook groups, focusing on topics related to products’ purchase, usage, reviews, etc. (e.g., groups concerned with personal care products, groups related to apparel products, etc.). We firstly looked up and identified a list of such Facebook groups with a large number of members. The targeted groups comprised between 10 and 200 thousand members each. Further on, we contacted group administrators, and asked for permission and support in disseminating the invitation to participate in our study. Eventually, we posted invitations in each group, including reminders, emphasizing the scientific and non-commercial scope of our investigation. 

The online questionnaire was developed using the Google Forms platform, and included two screening questions, as suggested by Simon and Tossan [3], in order to filter out respondents who did not access their Facebook account during the last month, as well as those who did not state their affinity for a brand by ‘Liking’ its page on Facebook. Further on, each eligible respondent was asked to fill in the questionnaire, considering a brand page that they were following on Facebook. 

After excluding straight liners, our sample included a total of 304 fully completed questionnaires. Most respondents referred to brands from the personal care industry (50.3%), the apparel industry (26.0%), or the food and beverage sector (7.6%). Considering the sample’s demographics, all respondents were adults, among which 32.2% between 18–24 years old, 45.1% between 25–34 years, 17.1% between 35–44 years, and 5.6% between 45–54 years. In 2021, the vast majority of Romanian Facebook users were adults from 18 to 54 years old, among which the largest age group was represented by users from 25 to 34 years old [42]. Thus, our investigated sample resembles, to a certain extent, the overall population of Facebook users in Romania.

To ensure that the sample did not exhibit any critical degree of non-response bias, we compared early and late respondents [43] in terms of demographic variables (i.e., age groups) using the Chi-square test. The results did not reveal any significant differences between early and late respondents, suggesting that there was no issue related to non-response bias.

### 3.2. Measurements

In order to assess social media page engagement, self-brand connection, and eWOM, we employed specific measurements, based on scales that were previously developed and validated in the literature. Considering their theoretical meaning and significance, all latent variables that were included in our research model were conceptualized as reflective.

To measure passive and active social media brand page engagement we adopted the scales derived by Simon and Tossan [3] from the consumer’s online brand-related activities (COBRA) framework [4,5]. Thus, we used three items to measure passive engagement, and another set of three items to evaluate active engagement, all answers being provided on a scale from 1 to 5 (at no time, once, 2–3 times, 4–5 times, more often). Particularly, we asked respondents to recall how often they had engaged with the referenced brand’s Facebook page, in various specific manners. For measuring self-brand connection and eWOM, we used four and three items, respectively, adopted from the scales proposed by Caroll and Ahuvia [20], with Likert answering options ranging from 1—strongly disagree, to 7—strongly agree. All items are outlined in detail in Table 1.

### 3.3. Data Analysis

Our proposed research model and hypothesized relationships were based on causal explanations and prediction. Additionally, the research was aimed not only at filling in literature gaps, but also at providing social media marketers with practical implications. Therefore, the PLS-SEM technique was considered the most appropriate for data analysis, as it provides a balance between explanation and prediction [44]. As for the software of choice, we employed SmartPLS 3, which provides all instruments needed to assess measurements, estimate model parameters, and evaluate its predictive relevance [45].

## 4. Results

### 4.1. Measurements Assessment

Before assessing the structural model and estimating model parameters, we evaluated the measurements in terms of convergent validity, internal consistency, and discriminant validity. 

For convergent validity and internal consistency assessment, we employed the criteria and thresholds suggested by Hair et al. [46]. As it can be seen in Table 1, all outer loadings have values above the cutoff point of 0.7, hence the results indicate appropriate indicator reliability for all items. Furthermore, as average variance extracted values go above the threshold value of 0.5 for all constructs, it can be stated that convergent validity is ensured for all latent variables. As for internal consistency, the PLS algorithm produced Cronbach’s Alpha and composite reliability values higher than the suggested minimum of 0.7. Consequently, it can be asserted that all four constructs in the model exhibit adequate internal consistency.

In order to evaluate the discriminant validity of our constructs we employed the heterotrait-monotrait ratio of correlations (HTMT) proposed by Henseler et al. [47]. We opted for this technique because previous studies [48,49] have indicated that the traditional Fornell-Larcker criterion for assessing discriminant validity is too liberal as compared to HTMT, and can lead to researchers overlooking potential discriminant validity issues. As it can be seen in Table 2, all HTMT values are much lower than the suggested threshold of 0.85, and, therefore, it can be stated that all four constructs included in our research model are conceptually distinct. 

### 4.2. Structural Model Assessment

Before the actual estimation of model parameters, each set of independent latent variables was checked for collinearity, to make sure that the estimated path coefficients using the PLS algorithm are not biased due to collinearity issues. As it can be seen in Table 3, variance inflation factor (VIF) values indicate no critical levels of collinearity, being considerably below the threshold of 3 [46]. 

To assess the structural model and estimate its parameters, we ran the PLS-SEM bootstrapping procedure with 5000 subsamples. The results are summarized in Table 4. As it can be seen, all research hypotheses included in our model are confirmed, the corresponding *p* values for all direct effects being below the significance threshold of 0.01. Additionally, the model’s coefficient of determination indicates a reasonable explanatory fit, with more than 36% of the variance in eWOM being explained by our model’s predictors (R^2^ = 0.362). Moreover, the results related to the indirect effects confirm the mediating role of self-brand connection in the relationship between social media brand page engagement and eWOM, for both passive and active engagement. 

Overall, our results suggest that more passive or active social media brand page engagement leads to more eWOM, as well as to better self-brand connection, which, in turn, leads to even more eWOM. Scrutinizing the results for total effects, it can be seen that the total impact (direct and indirect) of active social media brand page engagement on eWOM is slightly higher that the impact of passive engagement.

Additionally, we can emphasize a rather different mechanism of generating eWOM for the two types of social media brand page engagement. Thus, passive engagement has a considerably stronger direct influence on self-brand connection than on eWOM. On the other hand, active engagement is equally influential for both self-brand connection and eWOM. Yet, due to the mediating role of self-brand connection, the total impact of both active and passive engagement on eWOM is more or less the same. 

### 4.3. Predictive Power Assessment

Our research model and the hypothesized relationships were based not only on explanation, but also on prediction. Additionally, our goal was not only to fill in a literature gap, but also to provide practical implications for social media marketers. However, to ensure the meaningfulness and scientific soundness of such practical implications, the model’s predictive relevance needed to be confirmed [50].

To assess the model’s predictive power, we used the PLSPredict technique [50,51], which employs holdout samples and enables the computation of case-level predictions for each item of the target variable (i.e., eWOM). Following the procedure described by Shmueli et al. [50], we used 10 folds and 10 repetitions, and compared the RMSEs (root mean squared errors) produced by the PLS-SEM model with those obtained using a naïve linear model benchmark. Following the guidelines provided by Shmueli et al. [51], we concluded that the comparison of the RMSEs was feasible, as all indicators produced positive Q2_predict values. As it can be seen in Table 5, RMSEs obtained from the PLS-SEM prediction are lower than those generated by the naïve linear model benchmark, for all eWOM items. Hence, it can be asserted that our model has a high predictive power (i.e., passive and active social media brand page engagement, with the mediation of self-brand connection, can truly predict eWOM), and that any subsequent practical implication based on our results would be meaningful and scientifically sound. 

## 5. Discussion

From a theoretical perspective, our results are in line with previous knowledge regarding the relationship between social media brand page engagement, self-brand connection, and eWOM. The current paper confirms, for instance, that self-brand connection is an antecedent of word of mouth, as suggested by previous research [20,21,22] derived from the social identity theory [23]. Additionally, our results reinforce previous findings implying that more engagement with social media brand pages can lead to more eWOM [9,14], as well as to stronger self-brand connection [16,17,18,19], in line with the cultivation theory [24]. 

Nevertheless, the current paper depicts distinctively the impact of passive and active social media brand page engagement on eWOM, revealing an important mediating role of self-brand connection. Even though we find that both social media brand page consumption and contribution have a similar total impact on eWOM, we reveal a distinct process that leads to such an effect. While consumption or passive engagement primarily impacts self-brand connection, which further leads to eWOM, contribution or active engagements impacts eWOM in a more direct manner, self-brand connection having a weaker mediating role in this latter case. 

Additionally, the relatively similar impact of social media brand page consumption and contribution on eWOM needs to be emphasized as an important outcome of the current research. Despite active engagement being apparently more linked to consumers’ actions and behaviors, it does not have a considerably stronger total influence on eWOM than passive engagement. Even though passive engagement does not involve contributing any content to the brand’s social media page, it strongly impacts self-brand connection, which further leads to an actual online content contribution, via eWOM.

From a practical perspective, our results reveal important implications for social media marketers, emphasizing the importance of increasing the engagement of consumers with social media brand pages, in order to generate eWOM and, consequently, to attract new customers and to reinforce brand loyalty for existing ones.

More importantly, increasing social media brand page engagement in order to foster eWOM should not involve prioritizing active engagement before passive engagement (or vice versa), but rather investing in both directions, so as to generate both consumption of and contribution to the social media brand page.

There is plenty of research that can help practitioners understand what the drivers of consumers’ engagement with social media brand pages are. Capitalizing on the social exchange theory, various studies have emphasized several perceived benefits of social media brand pages that can truly enhance engagement. Among the most frequently emphasized benefits are interaction, information, and entertainment [9,14,52,53,54,55,56]. Previous studies have also emphasized remuneration [9,53,56], customization [52,54], and identification [9,56] as important benefits that, if perceived as such, can enhance consumers’ engagement with social media brand pages.

Therefore, a combination of compelling visual and textual content, which should address consumers’ interests and preferences, or their comments or questions posted in social media, has the potential to stimulate interaction with the social media content created by brands [57]. 

To generate engagement, social media marketers should manage to create content that is entertaining, enjoyable, fun, but also practical, useful and helpful, as such content will encourage consumers not only to consume brand-related content, but also to take one step further and to contribute with content [14,52,54,55,58]. Vivid posts should include photos, videos, animation, links, quizzes/polls, or click-throughs to ensure a large dissemination, to facilitate interaction with the brand, and also to stimulate content sharing by users [17,52,58,59]. 

One popular way for brands to provide consumers with entertainment, fun and pleasure in social media is gamification, sometimes referred to as advergaming or advertainment [60,61]. Gaming features generate high levels of active engagement and increase the willingness of consumers to interact [60,62], this leading to a more powerful identification with the brand [63] and contributing to users’ immersion and community experience [55].

Additionally, information posted in social media by brands should be perceived by consumers as captivating and interesting [54,55], novel and dynamic [17], as this will generate consumers’ attention and stimulate involvement with the brand-related content.

It is also very important to offer consumers the possibility to interact with the brand, or with other consumers on social media brand pages. This can be accomplished through discussing product usage, providing product comparisons [52], offering brand-related information [51], requesting inputs from users, replying to consumers’ comments, encouraging consumers to generate content, and publicly recognizing it and even repost it as this will satisfy consumers’ self-expressiveness needs [9]. 

Posting messages that create a sense of belonginess, creating a community around a brand page will stimulate the development of an emotional link between consumers and the brand, and will conversely lead to higher levels of interaction, collective cooperation, and collaboration [9,14,59]. Social media marketers should make the consumers feel important for the brand community and should stimulate them to contribute with ideas, concerns, experiences, as this will increase their feelings of community and belonging to the brand community [62].

## 6. Conclusions

Our results show that social media brand page engagement, either passive or active, has a positive impact on eWOM, both directly and indirectly, via self-brand connection. In other words, more social media brand page consumption (page visits, posts reading, pictures or videos viewing, etc.) or contribution (commenting on posts, photos or videos, asking questions, engaging in conversations the page community, etc.) leads to stronger self-brand connection and, consequently, to more eWOM. 

Additionally, our research reveals that the two types of social media brand page engagement generate eWOM distinctly: although passive engagement has a considerably stronger direct influence on self-brand connection than on eWOM, active engagement is equally influential for both self-brand connection and eWOM. However, due to the mediating role of self-brand connection, the total effect on eWOM is relatively equal for both passive and active engagement.

Our findings provide practical implications for social media marketers, emphasizing the importance of increasing the engagement of consumers with social media brand pages, in order to generate eWOM and, consequently, to attract new customers and to reinforce brand loyalty for existing ones.

Our study exhibits several limitations, of which several represent opportunities for future research. 

Firstly, our data collection span was limited to Facebook and to Romanian Facebook users. Even though our findings can be, to a certain extent, applied to other social media platforms and/or to users from other geographical areas, future research should investigate the proposed structural model within other social media, which are gaining in popularity, as well as among users from geographical areas with significantly different cultural characteristics. 

Secondly, our model does not take into account the potential moderating role of consumers’ psychographic characteristics. Previous studies (e.g., [31]) have suggested that personality traits (e.g., extraversion, self-esteem, etc.) have a significant impact on how consumers use social media, and on how they relate to brands within social networking platforms. Hence, future research should be conducted to investigate the moderating role of various consumers’ personality traits in the relationship between social media brand page engagement and eWOM.

Thirdly, it is worth pointing out that our study focuses exclusively on generating positive eWOM, as a result of social media brand engagement and self-brand connection. On the other hand, negative eWOM plays an important role for both customer retention and acquisition. Social media pages allow consumers to express not only positive brand-related experiences, but also negative ones. Future research should also investigate whether such negative posts/comments have a significant impact on self-brand connection, and whether they generate negative eWOM. Such research could provide valuable insights for managing and confining negative eWOM.

Last, but not least, our study is based on a small sample (N = 304), selected non-randomly. Even though our sample resembles, to a certain extent, the investigated population (at least in terms of age groups), the convenience sampling might cast doubts on its adequacy for statistical inference. Nevertheless, the data analysis method used in this study (PLS-SEM) enables complex models to be estimated with adequate statistical power even when small samples are involved [64]. For instance, considering the R^2^ value for our model’s target variable (0.362), the number of predictors involved in the model (3), the sample size, and a *p*-value of 0.05, the resulting post hoc statistical power is 1.0, which is significantly larger than the standard threshold of 0.8 [46]. Moreover, if we used the less liberal inverse square root method for assessing the adequacy of a sample size for PLS-SEM [46], considering the value of the path coefficient with the minimum magnitude (0.168), a common statistical power level of 0.8, and a *p*-value of 0.05, the minimum required sample size would be (2.486/0.168)^2^ = 219. As it can be seen, our sample is significantly larger than this minimum threshold.

## Figures and Tables

**Table 1:** Internal consistency and convergent validity assessment.

Latent Variables	Items	OL	CA	CR	AVE
Passive social media brand page engagement	During the last month, how often did you …				
	visit the page?	0.858	0.852	0.910	0.771
	read page posts?	0.886			
	view pictures or videos uploaded on the page?	0.891			
Active social media brand page engagement	During the last month, how often did you …				
	comment on page posts, photos or videos?	0.783	0.805	0.886	0.722
	ask the page community questions?	0.868			
	engage in conversations with the page community?	0.894			
Self-brand connection	This brand symbolizes the kind of person I really am inside	0.910	0.946	0.961	0.860
	This brand reflects my personality	0.922			
	This brand is an extension of my inner self	0.937			
	This brand mirrors the real me	0.940			
Electronic word of mouth	During the last month, I …				
	recommended this brand to my friends on the Internet	0.965	0.964	0.977	0.933
	spread good words on the Internet about this brand	0.969			
	gave this brand lots of positive word of mouth on the Internet	0.964			

Abbreviations: OL = Outer loadings; CA = Cronbach’s Alpha; CR = Composite Reliability; AVE = Average Variance Extracted.

**Table 2:** Discriminant validity assessment.

	Active SMBPE	Passive SMBPE	Self-Brand Connection
Passive SMBPE	0.501		
Self-brand connection	0.424	0.453	
eWOM	0.497	0.456	0.544

Note: Values represent heterotrait-monotrait ratios of correlations (HTMT values). Abbreviations: SMBPE = Social media brand page engagement.

**Table 3:** Collinearity assessment.

Predictors of Self-Brand Connection	VIF	Predictors of eWOM	VIF
Passive SMBPE	1.212	Passive SMBPE	1.337
Active SMBPE	1.212	Active SMBPE	1.285
		Self-brand connection	1.278

Abbreviations: VIF = Variance inflation factor; SMBPE = Social media brand page engagement.

**Table 4:** Structural model assessment.

	**Effect**	***p* Value**
**Direct effects**		
*H1:* Self-brand connection → eWOM	0.364	0.000
*H2a:* Passive SMBPE → Self-brand connection	0.313	0.000
*H2b:* Active SMBPE → Self-brand connection	0.239	0.000
*H3a:* Passive SMBPE → eWOM	0.168	0.002
*H3b:* Active SMBPE → eWOM	0.234	0.000
**Indirect effects**		
Passive SMBPE → Self-brand connection → eWOM	0.114	0.000
Active SMBPE → Self-brand connection → eWOM	0.087	0.000
**Total effects**		
Passive SMBPE → eWOM	0.281	0.000
Active SMBPE → eWOM	0.321	0.000

Note: PLS-SEM bootstrapping procedure with 5000 subsamples. Abbreviations: SMBPE = Social media brand page engagement.

**Table 5:** Structural model predictive power assessment for eWOM as target variable.

Items	Q^2^_Predict_pls_	RMSE_PLS_	RMSE_LM_	RMSE_PLS_ < RMSE_LM_
eWOM1	0.226	1.894	1.910	Yes
eWOM2	0.237	1.888	1.900	Yes
eWOM3	0.232	1.842	1.856	Yes

Note: PLSPredict procedure with 10 folds and 10 repetitions. Abbreviations: RMSE_PLS/LM_ = root mean squared error of prediction using PLS-SEM/using a naïve linear model.

## Data Availability

The data that support the findings of this study are available from the corresponding author upon reasonable request.
